# The Mechanism of Two Benzaldehydes from *Aspergillus terreus* C23-3 Improve Neuroinflammatory and Neuronal Damage to Delay the Progression of Alzheimer’s Disease

**DOI:** 10.3390/ijms24020905

**Published:** 2023-01-04

**Authors:** Minqi Chen, Jinyue Liang, Yi Liu, Yayue Liu, Chunxia Zhou, Pengzhi Hong, Yi Zhang, Zhong-Ji Qian

**Affiliations:** 1 College of Food Science and Technology, School of Chemistry and Environment, Guangdong Provincial Key Laboratory of Aquatic Product Processing and Safety, Guangdong Province Engineering Laboratory for Marine Biological Products, Shenzhen Insititute of Guangdong Ocean University, Guangdong Ocean University, Zhanjiang 524088, China; 2 Southern Marine Science and Engineering Guangdong Laboratory, Zhanjiang 524000, China; 3 Collaborative Innovation Center of Seafood Deep Processing, Dalian Polytechnic University, Dalian 116034, China

**Keywords:** benzaldehydes, *Aspergillus terreus* C23-3, Alzheimer’s disease, BV-2, HT-22

## Abstract

Alzheimer’s disease (AD), a neurodegenerative disease, is the most common cause of dementia in humans worldwide. Although more in-depth research has been carried out on AD, the therapeutic effect of AD is not as expected, and natural active substances are increasingly sought after by scientists. In the present study, we evaluated two benzaldehydes from a coral-derived *Aspergillus terreus* strain C23-3, their anti-neuroinflammatory activity in microglia (BV-2), and their neuroprotective activity and mechanisms in hippocampal neuronal cells (HT-22). These include the protein expression of iNOS, COX-2, MAPKs pathways, Tau protein-related pathways, caspases family-related signaling pathways. They also include the levels of TNF-α, IL-6, IL-18 and ROS, as well as the level of mitochondrial oxidative stress and neuronal cell apoptosis. The results showed that both benzaldehydes were effective in reducing the secretion of various inflammatory mediators, as well as pro-inflammatory factors. Among these, benzaldehyde **2** inhibited mitochondrial oxidative stress and blocked neuronal cell apoptosis through Tau protein-related pathways and caspases family-related signaling pathways, thereby inhibiting β-amyloid (Aβ)-induced neurological damage. This study reveals that benzaldehyde **2** has potential as a therapeutic agent for Alzheimer’s disease, and offers a new approach to the high-value use of marine natural products.

## 1. Introduction

Alzheimer’s disease (AD), a neurodegenerative disease, is the most common cause of dementia in humans worldwide [1,2,3]. As the average life expectancy increases, AD is predicted to impact 131.5 million people globally by 2050 [4]. AD has a number of histological features, including the formation of large patches of extracellular β-amyloid (Aβ) in the brain, as well as hyperphosphorylation of the microtubule-associated protein tau within neurons, causing neurogenic fibrillary tangles (NFT) [1,2,3]. This, in turn, leads to neuronal dysfunction and cell death.

Tau, as a major component of NFT within neurons in the context of AD, is a microtubule-associated protein (MAP). In the central nervous system (CNS), tau proteins bind to axonal microtubules to form stable structures under normal circumstances; however, this homeostasis is altered when AD occurs [5,6]. In the context of AD, the activities of GSK-3α/β and CDK5, the kinases responsible for phosphorylation of tau, are altered. This is followed by hyperphosphorylation of tau, leading to impaired microtubule stabilization and an absence of tau in the microtubules [7]. Microtubule de-stabilization and tau oligomerization eventually lead to neurofibrillary tangles in the cell, which further lead to neuronal apoptosis [5].

Mitochondrial dysfunction is an early feature of AD, including impairment of energy metabolism, defects of activity and function of key respiratory enzymes, accumulation and generation of mitochondrial reactive oxygen species and formation of membrane permeability transition pores [8,9]. Mitochondria may be vital players in Aβ-induced neuronal energy damage and oxidative damage, which play a critical role in neuronal perturbation and necrotic and apoptotic cell death in AD [8]. When mitochondrial function is impaired and the mitochondrial membrane potential (Δψm) is reduced or even lost, cytochrome C moves from the mitochondria to the cytoplasm [10]. Cytochrome C release is a key step in cell apoptosis, triggering a cascade of caspase activation that leads to cell death.

Another histological feature of AD is the accumulation of astrocytes and microglia around plaques [11,12], collectively referred to as ‘neuroinflammation’, but more properly classified as astrocyte hyperplasia and microgliosis [13,14,15]. Microglia, a key factor in the pathogenesis of AD, are a self-renewing population of myeloid cells that function as innate immune cells in the brain [16]. Thus, under stress conditions, they may produce various mediators such as inducible nitric oxide synthase (iNOS) and cyclooxygenase-2 (COX-2), tumor necrosis factor-α (TNF-α), interleukin-18 (IL-18), interleukin-6 (IL-6) and some chemokines that cause neuronal damage, among others [17].

Although understanding of AD continues to improve, its exact etiology and pathogenesis remain unclear. Current AD treatments are not as effective as they should be, and some drugs have strong side effects. Secondary metabolites of marine fungi have become increasingly popular among scientists in recent years, due to their diverse activities and novel structures. In previous studies, we have isolated several compounds with antioxidant, anti-inflammatory and anti-acetate cholinesterase activities from a coral-derived *Aspergillus terreus* strain C23-3 [18,19,20,21]. Benzaldehyde is a common natural product of *Aspergillus* spp., *Chae tomium* sp. and *Eutotium* sp. These compounds usually exhibit significant biological activities such as antibacterial, anti-inflammatory, antitumor, cytotoxic and antioxidant activities [22,23,24,25]. Therefore, they are of increasing interest to scientists. In these studies, we used LPS-induced BV-2 and Aβ–induced HT-22 as models to explore, in depth, the potential effects and mechanisms of action of two benzaldehyde analogues isolated from *Aspergillus terreus* strain C23-3 on neuroinflammation and Alzheimer’s disease.

## 2. Results

### 2.1. Cytotoxic Assessment

**Figure 1:**
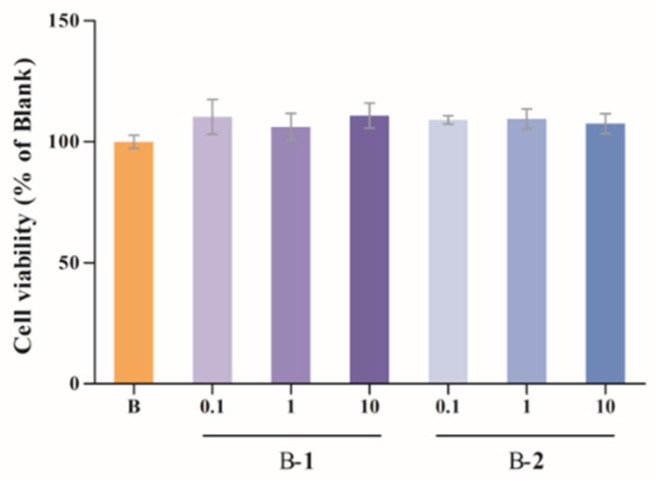
The cell viability of benzaldehydes **1**–**2** in BV-2 cells. Data were expressed as the means ± S.D (*n* = 3).

In order to ascertain whether two benzaldehydes (**1** and **2**) exerted cytotoxicity on BV-2 cells, a CCK-8 assay was performed. As can be seen in Figure 1, the cell survival rate of the B–**1** and B–**2** groups was not significantly different from that of the blank group, indicating that the various concentrations (0.1, 1 and 10 μM) of two benzaldehydes (**1** and **2**) were not cytotoxic to BV-2 cells. Therefore, these concentrations were used for further investigation.

### 2.2. Effect of Two Benzaldehydes (***1*** and ***2***) on Levels of IL-6, TNF-α and IL-18

**Figure 2:**
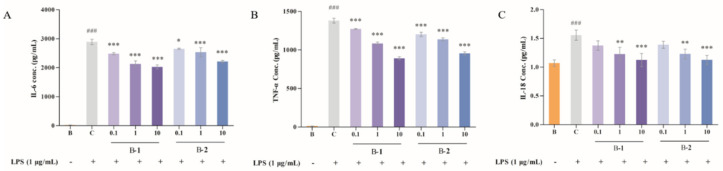
(**A**) IL-6 level of BV-2 cells treated by benzaldehydes **1**–**2**. (**B**) TNF-α level of BV-2 cell treated by benzaldehydes **1**–**2**. (**C**) IL-18 level of BV-2 cell treated by benzaldehydes **1**–**2**. Data are expressed as the means ± SD (*n* = 3). ### *p* < 0.001 vs. normal control group, * *p* < 0.05, ** *p* < 0.01, and *** *p* < 0.001 vs. LPS induced group.

Secretions of IL-6, TNF-α and IL-18 were detected by ELISA kits. As shown in Figure 2, after LPS induction, IL-6, TNF-α and IL-18 levels were increased from 22.18 to 2890.64, 11.38 to 1380.87 and 1.07 to 1.56, respectively. After treatment with either of two benzaldehydes (**1** and **2**) in various concentrations (0.1, 1 and 10 μM), secretion levels of IL-6, TNF-α and IL-18 were significantly reduced, which was a concentration–dependent result. 

### 2.3. Effect of Two Benzaldehydes (***1*** and ***2***) on Expression of iNOS, COX-2 and MAPKs Signaling Pathways

**Figure 3:**
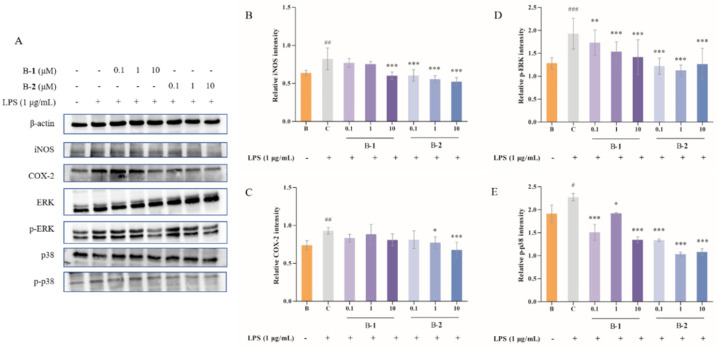
(**A**) Immunoblotting results of iNOS, COX-2, ERK, p-ERK, p38 and p-p38. (**B**) iNOS production of BV-2 cell treated by benzaldehydes **1**–**2**. (**C**) COX-2 production of BV-2 cell treated by benzaldehydes **1**–**2**. (**D**) p-pERK production of BV-2 cell treated by benzaldehydes **1**–**2**. (**E**) p-p38 production of BV-2 cell treated by benzaldehydes **1**–**2**. Cell lysates, antibodies against iNOS, COX-2, ERK, p-ERK, p38 and p-p38, were used to conduct Western blot analysis. β-actin was used as an internal control. Data are expressed as the means ± SD (*n* = 3). # *p* < 0.05, ## *p* < 0.01, and ### *p* < 0.001 vs. normal control group, * *p* < 0.05, ** *p* < 0.01, and *** *p* < 0.001 vs. LPS induced group.

Release and expression levels of inflammatory cytokines (iNOS and COX-2) are markers of inflammation and oxidative stress. Their expression in BV-2 cells was determined by the protein level using Western blotting (Figure 3A–C). The expression of iNOS and COX-2 was enhanced by exposure to LPS for 24 h, but relieved from these after being treated with Benzaldehyde **2**. However, Benzaldehyde **1** inhibited the iNOS level in the high dose group (10 μM), but had no obvious effect, in any of the dose groups, on the expression of COX-2. It is clear that the ability of Benzaldehyde **2** to inhibit iNOS and COX-2 protein expression is stronger.

The MAPKs signaling pathway, usually stimulated by growth factors and cellular stress or inflammatory cytokines, plays a signal transduction role in various chronic diseases. As illustrated in Figure 3D,E, after LPS stimulation, the protein secretion of p-ERK and p-p38 was increased significantly, rising to 1.93 and 2.27, respectively. However, the phosphorylation of ERK and p38 was alleviated by treatment with two benzaldehydes (**1** and **2**) at different concentrations. Among these, the effect was more pronounced in the B–**2** groups compared with the control group, with a maximum reduction of 41.45% and 54.63%, respectively. Benzaldehyde **2** was more effective than benzaldehyde **1** in reducing the protein secretion of p-ERK and p-p38. Based on the above results, benzaldehyde **2** was used in subsequent experiments for further study.

### 2.4. Effect of Benzaldehydes ***2*** on ROS Production

**Figure 4:**
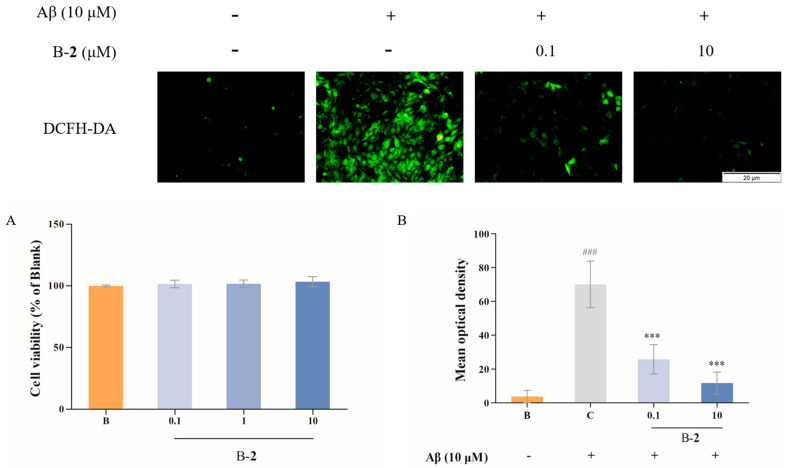
(**A**) The cell viability of compound **2** in HT-22 cells. (**B**) ROS level of benzaldehydes **2** in HT-22 cells. Data are expressed as the means ± SD (*n* = 3). ### *p* < 0.001 vs. normal control group, *** *p* < 0.001.

Firstly, a toxicity test was performed to measure the effect of benzaldehydes **2** in HT-22, and the result is shown. The results showed that the two concentrations (0.1 and 10 μM) of benzaldehyde **2** had no significant effect on the survival rate of the cells (Figure 4A). Thus, the two concentrations were further used in the subsequent experiments. 

In order to determine whether benzaldehyde **2** would have an effect on the production of ROS in HT-22 cells, a fluorescence experiment was performed. As could be found in Figure 4B, green fluorescence intensity in HT-22 cells was enhanced by adding Aβ 25–35, whereas the treatment of two concentrations of benzaldehyde **2** (0.1 and 10 μM) was obviously decreasing. This indicates that both of 0.1 and 10 μM of benzaldehyde **2** could clearly suppress the production of ROS.

### 2.5. Effect of Benzaldehydes ***2*** on Tau Related Pathway

**Figure 5:**
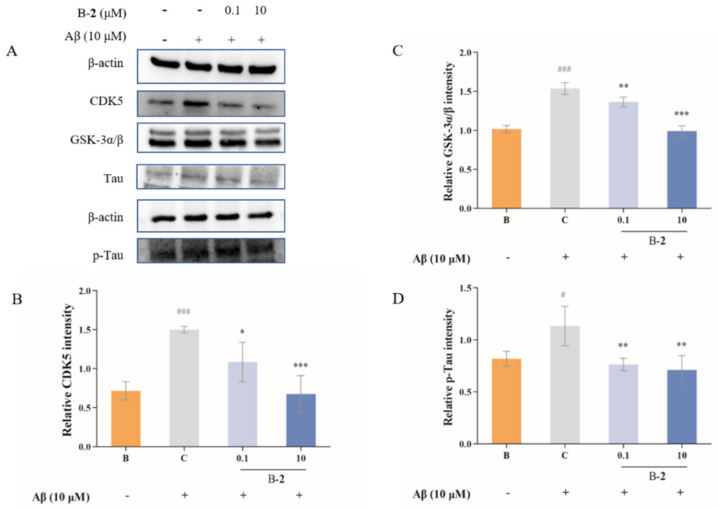
(**A**) Immunoblotting results of CDK5, GSK-3α/β, Tau and p-Ta. (**B**) CDK5 production of HT-22 cells treated by benzaldehydes **2**. (**C**) GSK-3α/β production of HT-22 cells treated by benzaldehydes **2**. (**D**) p-Tau production of HT-22 cell treated by benzaldehydes **2**. Cell lysates, antibodies against CDK5, GSK-3α/β, Tau and p-Tau were used to conduct Western blot analysis. β−actin was used as an internal control. Data are expressed as the means ± SD (*n* = 3). # *p* < 0.05 and ### *p* < 0.001 vs. normal control group, * *p* < 0.05, ** *p* < 0.01, and *** *p* < 0.001 vs. LPS induced group.

GSK-3α, GSK-3β and CDK5 are kinases primarily responsible for phosphorylation of tau, which, in turn, leads to dissociation of tau from microtubules. As shown in Figure 5, the results revealed that the expression level of CDK5, GSK-3α and GSK-3β obviously increased, and tau was phosphorylated in Aβ-stimulated cells. This upgradation in cells was slightly inhibited by 0.1 μM benzaldehyde **2** pretreated for 48 h, from 1.50 ± 0.05 to 1.09 ± 0.33, 1.54 ± 0.09 to 1.37 ± 0.08 and 1.13 ± 0.20 to 0.76 ± 0.06, respectively. Treatment with 10 μM of benzaldehyde **2** significant reduced the CDK5, GSK-3α and GSK-3β in the Aβ-stimulated groups (Figure 5). Finally, 10 μM treated with benzaldehyde **2** expressively downregulated the phosphorylated levels of p-Tau, and reduced more than 37% of the Aβ-stimulated group. The above results indicate that benzaldehyde **2** treatment obviously suppressed the tau-related signaling pathway. 

### 2.6. Effect of Benzaldehyde ***2*** on Mitochondrial Membrane Potential

**Figure 6:**
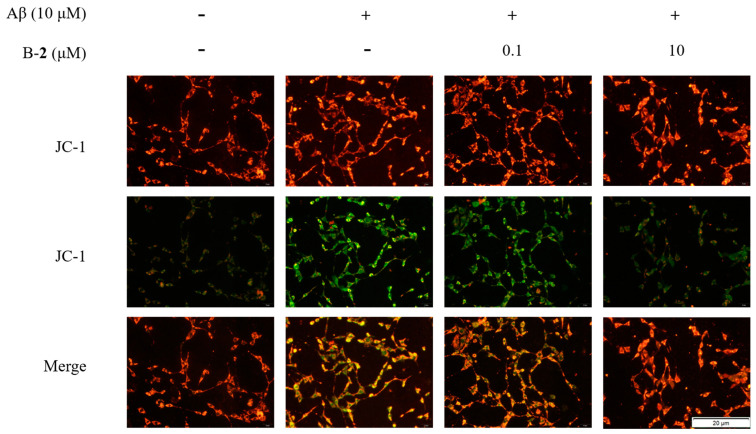
Mitochondrial membrane potential of HT-22 cell treated by benzaldehydes **2**. Data are expressed as the means ± SD (*n* = 3).

Oxidative stress in mitochondria leads to changes in membrane potential. In a fluorescence assay, the Aβ-stimulated group exhibited an obvious mean optical density of green fluorescence in contrast to the blank group (Figure 6), indicating a decrease in mitochondrial membrane potential. After treatment with 0.1 μM or 10 μM of benzaldehyde **2**, the green fluorescence intensity distinctly weakened, and red fluorescent predominantly indicated that mitochondrial membrane potential was up-regulating (Figure 6). The above results indicated that the addition of benzaldehyde **2** significantly alleviated the decrease in mitochondrial membrane potential. 

### 2.7. Effect of Benzaldehydes ***2*** on Expression of Cytochrome C and Caspase Family Related Pathway

**Figure 7:**
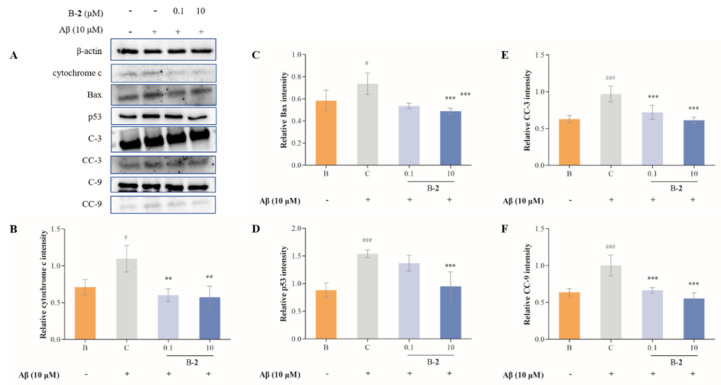
(**A**) Immunoblotting results of cytochrome c, Bax, p53, caspase-3, cleaved caspase-3, caspase-9 and cleaved caspase-9. (**B**) cytochrome c production of HT-22 cells treated by benzaldehydes **2**. (**C**) Bax production of HT-22 cells treated by benzaldehydes **2**. (**D**) p53 production of HT-22 cells treated by benzaldehydes **2**. (**E**) cc-3 production of HT-22 cells treated by benzaldehydes **2**. (**F**) cc-9 production of HT-22 cells treated by benzaldehydes **2**. Cell lysates, antibodies against cytochrome c, Bax, p53, caspase-3, cleaved caspase-3, caspase-9 and cleaved caspase-9 were used to conduct Western blot analysis. β−actin was used as an internal control. Data are expressed as the means ± SD (*n* = 3). # *p* < 0.05 and ### *p* < 0.001 vs. normal control group, ** *p* < 0.01, and *** *p* < 0.001 vs. LPS induced group.

The release of mitochondrial cytochrome C plays an important role in the process of apoptosis, and its release into the cytoplasm triggers the activation of caspase cascade, leading to cell death. The release of cytochrome C is the result of increased permeability of the outer mitochondrial membrane. Release and expression levels of cytochrome C were dramatically improved in the Aβ-induced HT-22 cells compared to the untreated cells (Figure 7A,B). However, this increase was reversed by the addition of benzaldehydes **2**. 

The results show that the protein levels of Bax, p53, cleaved caspase-9 (CC-9) and cleaved caspase-3 (CC-3) were increasing in the control group in contrast to the blank group, from 0.58 ± 0.13 to 0.74 ± 0.13, 0.88 ± 0.18 to 1.54 ± 0.08, 0.64 ± 0.09 to 1.00 ± 0.24 and 0.63 ± 0.10 to 0.97 ± 0.24, respectively (Figure 7). This increase was modified by the addition of benzaldehyde **2**. Treatment of 0.1 μM of benzaldehyde **2** slightly inhibited the expression of Bax, but it does not affect p53. The addition of higher dose of benzaldehyde **2** (10 μM) clearly suppressed their production, and the levels decreased to 0.49 ± 0.03 and 0.95 ± 0.34, respectively. At the same time, there were a significant decrease in both CC-3 and CC-9 protein levels in all concentrations of B-**2** groups, in a concentration-dependent pattern. The results showed that 10 μM of benzaldehyde **2** reduced 36.71% of CC-3 expression and 44.63% of CC-9 expression.

### 2.8. Effect of Benzaldehyde ***2*** on Cell Cycle and Apoptosis

**Figure 8:**
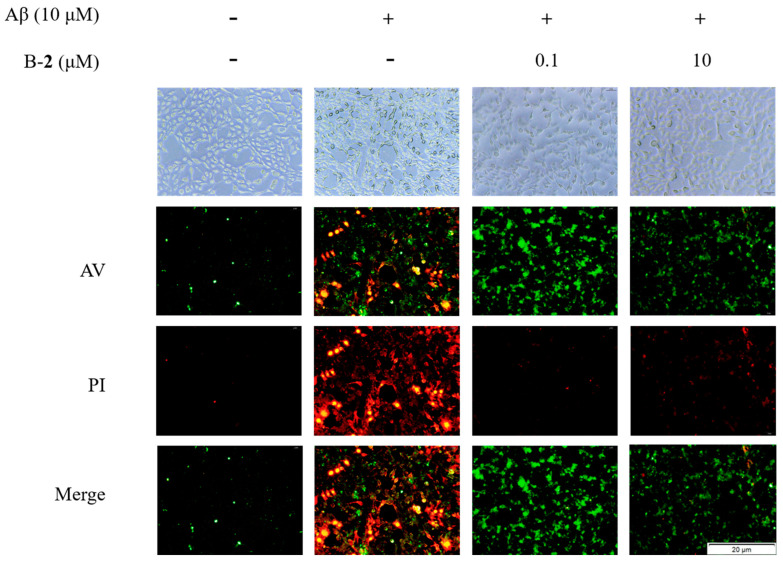
Cell cycle and apoptosis of HT-22 cells treated by benzaldehydes **2**. Data are expressed as the means ± SD (*n* = 3).

To determine the effect of benzaldehyde on Aβ-induced HT-22 cell cycle and apoptosis, we performed a fluorescence assay using the cell cycle and apoptosis analysis kit. As can be found in Figure 8, weak red and green fluorescence was observed in unstimulated cells, while stronger red and green fluorescence was observed after Aβ stimulation. This indicated a significant increase in the number of apoptotic cells in both early and mid-late stages. Compared to the Aβ-stimulation group, the red fluorescence was significantly lower in the 0.1 μM group, and the green fluorescence was also slightly lower, indicating that the number of apoptotic cells in the middle and late stages was reduced, and that most of the apoptotic cells were in the early stage. In the 10 μM group, only faint red and green fluorescence could be observed, indicating that only very few apoptotic cells were present at that time. 

### 2.9. Docking Study of Cytochrome C with Benzaldehyde ***2***

**Figure 9:**
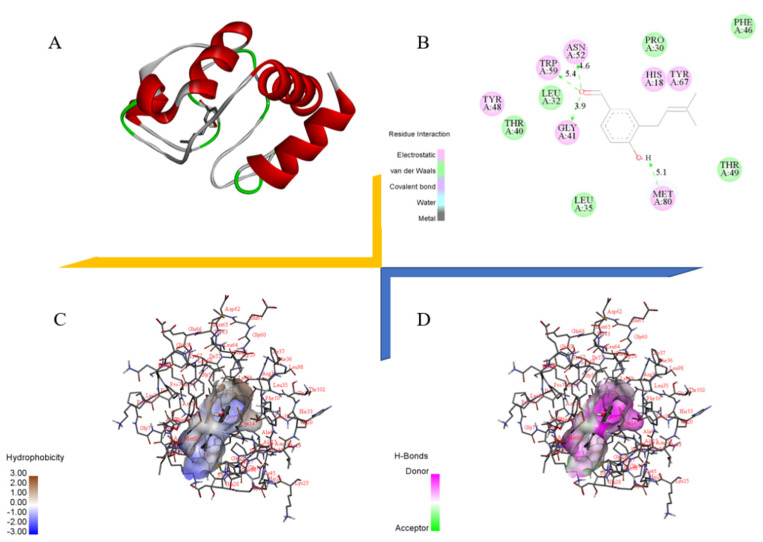
Docking study of Cytochrome−C with benzaldehydes **2**. (**A**) Docking study of Cytochrome C with benzaldehydes **2**. (**B**) Important residue formed between Cytochrome C with benzaldehydes **2**. (**C**) Hydrophobic interaction of Cytochrome C with benzaldehydes **2**. (**D**) Hydrogen-bond interaction of Cytochrome C with benzaldehydes **2**.

In order to elucidate the mode of action of cytochrome C with benzaldehyde **2** at the molecular level, molecular docking was performed. Benzaldehyde **2** was docked to the active pocket of cytochrome C with an affinity of −7.2 kcal/mol, and the theoretical binding pattern is shown in Figure 9. As can be seen from Figure 9A, benzaldehyde **2** showed a compact binding pattern in the active pocket. Benzaldehyde **2** was found in a luminal pocket composed of amino acids His-18, Pro-30, Leu-32, Leu-35, Thr-40, Gly-41, Phe-46, Tyr-48, Thr-49, Asn-52, Trp-59, Tyr-67 and Met-80, forming a strong hydrophobic interaction (Figure 9B,C). Importantly, benzaldehyde **2** can form hydrogen bonding interactions with amino acids Gly-41, Asn-52, Trp-59 and Met-80, with lengths of 3.9 Å, 4.6 Å, 5.4 Å and 5.1 Å, respectively (Figure 9B,D).

## 3. Discussion

Marine fungi have proven to be a major source of marine natural products in recent years. *Aspergillus terreus* belongs to *Aspergillus,* in the Trichocomaceae family of Eurotiales, in the Eurotiomycetes of the Ascomycota phylum. It has been reported that its secondary metabolites contain rich and diverse compounds, such as butyrolactones, benzaldehydes, territrem, etc. [18,26]. In this study, the effects of two benzaldehydes isolated from *Aspergillus terreus* C23-3 on neuroinflammation and nerve injury, as well as their related mechanisms were evaluated by two cell lines.

Neuroinflammation regulated by microglia is responsible for neurodegenerative diseases. Microglia can perform neuroprotective and neurotoxic functions in the brain. Therefore, inhibition of microglia hyperactivation is an effective way to control neuroinflammation [27]. After activation of external stimulation, microglia release proinflammatory factors and cytokines, such as iNOS, COX-2, TNF-α, IL-6 and IL-18, etc., promote the processing and synthesis of amyloid precursor protein, leading to neuronal death. These events are thought to be hallmarks of neuroinflammation in the pathological process of neurodegenerative diseases [28]. Therefore, reducing microglia activation is beneficial in the prevention or treatment of neurodegenerative diseases [29]. MAPK, which includes c-Jun N-terminal kinase (JNK), extracellular signal-related kinase (ERK) –1/2 and p38, is the classic inflammation–related signaling pathway. Regarding the compound structure, benzaldehyde **1** and benzaldehyde **2** contain three and one phenolic groups, respectively. Phenolic compounds have been proven to have good antioxidant and anti-inflammatory effects [30]. Both benzaldehyde **1** and benzaldehyde **2** can exhibit a compact binding mode in the active pocket of iNOS. Benzaldehyde **1** is found in a cavity pocket composed of amino acids Trp-188, Cys-194, Leu-203, Ser-236, Ile-238, Pro-344, Val-346, Phe-363, Asn-364, Gly-365, Trp-366 and Tyr-483, while benzaldehyde **2** is found in a pocket consisting of amino acids Trp-188, Ala-191, Cys-194, Leu-203, Ser-236, Ile-238, Met-349, Phe-363, Asn-364, Gly-365 and Tyr-483. A strong hydrophobic interaction is formed. Importantly, benzaldehyde **1** can form a hydrogen bond with amino acid Tyr-483 at a length of 5.8 Å, and can form π–π bonds with amino acids Trp-188 and Phe-363, respectively. Benzaldehyde **2** can form a hydrogen bond with amino acid Tyr-483, of 5.7 Å length. Both benzaldehyde **1** and benzaldehyde **2** can exhibit a compact binding pattern in the active pocket of COX-2, forming strong hydrophobic interactions. In addition, benzaldehyde **1** can form hydrogen bond interactions with amino acids Tyr-341 and Phe-504, with lengths of 4.7 Å and 3.4 Å, respectively, and forms π–π bonds with amino acid Ser-339. Benzaldehyde **2** can form hydrogen bonds 3.9 Å, 6.4 Å and 4.5 Å, along with amino acids Tyr-371, Met-508 and Ser-516, respectively [18]. The results showed that both benzaldehydes were effective in reducing the protein secretion of iNOS, COX-2, TNF-α, IL-6 and IL-18 in LPS-stimulated BV-2 cells, thus acting as a neuroinflammatory agent (Figure 2 and Figure 3A–C). Western blot results also showed that both types of benzaldehyde inhibited MAPKs signaling pathways by reducing the protein levels of p-ERK, p-JNK and p-p38 (Figure 3D,E), thereby preventing and treating neuroinflammation.

The amyloid cascade hypothesis suggests that dysregulated amyloid processing or abnormal accumulation of beta-amyloid peptides is a potential trigger for subsequent pathophysiological events in the later stages of AD, including the formation of NFTs. Intracellular aggregates of tau, in either soluble or insoluble forms, disrupt cellular mechanisms and synaptic function, ultimately causing neurofibrillary tangles and leading to neuronal death, as well as deposition of insoluble aggregates of the microtubule-binding protein tau [31]. Phosphorylation has been extensively studied and is commonly found on tau proteins. Kinases, such as CDK5 and GSK-3α/β, maintain and regulate tau phosphorylation in an overlapping manner, allowing multiple kinases to phosphorylate tau at a given locus. Aberrant activation of CDK5 acts early in neuronal death, even before mitochondrial dysfunction. Therefore, maintaining CDK5 homeostasis is considered a suitable therapeutic target for improving AD pathological processes, such as neuronal apoptosis and tau pathology [30,31,32]. In addition to phosphorylation, more than 100 potential post-translational modification sites have been observed on tau proteins [29,30,31,32]. Any alteration in the normal expression of tau isoforms may lead to aggregation. The results of this study showed that benzaldehyde **2** could effectively reduce the activity of CDK5 and GSK-3α/β, thus hindering the promotion of tau protein phosphorylation by CDK5 and GSK-3α/β and maintaining tau protein microtubule homeostasis (Figure 5). In addition, the results of Western blot and fluorescence experiments illustrated that benzaldehyde **2** could also directly reduce the degree of tau protein phosphorylation, and thus inhibit the apoptosis of neuronal HT-22 cells.

Imbalance in mitochondrial functioning in neuronal cells triggers mitochondrial dysfunction, which subsequently leads to reduced mitochondrial membrane potential, ATP depletion, ROS accumulation and increased apoptosis [33,34,35]. During cell death, various proteins normally isolated in the intermembrane space of mitochondria, including cytochrome C, apoptosis-inducing factors and certain proteasome proteins, are released into the cytoplasm [36]. In the cytosol, cytochrome C activates the caspases, a family of killer proteases, through formation of a complex with procaspase-9 [36]. Caspase activation is a principal stage of apoptosis cascade [36]. The experimental results showed that the addition of benzaldehyde **2** effectively inhibited the production of ROS (Figure 4B), the mitochondrial membrane (Figure 6) potential rebounded, the mitochondrial oxidative stress was improved and the overflow of cytochrome C was alleviated (Figure 7). Benzaldehyde **2** is found in a luminal pocket composed of amino acids His-18, Pro-30, Leu-32, Leu-35, Thr-40, Gly-41, Phe-46, Tyr-48, Thr-49, Asn-52, Trp-59, Tyr-67 and Met-80, forming a strong hydrophobic interaction (Figure 9B,C). Further, benzaldehyde **2** formed hydrogen bonding interactions with amino acids Gly-41, Asn-52, Trp-59 and Met-80, with lengths of 3.9 Å, 4.6 Å, 5.4 Å and 5.1 Å, respectively (Figure 9B,D). This is the predominant force between benzaldehyde **2** and cytochrome C. These interactions allow benzaldehyde **2** and cytochrome C to form stable complexes. The molecular docking studies described above give a reasonable explanation for the interaction between benzaldehyde **2** and cytochrome C, and provide a basis for further studies on the mechanism of interaction between benzaldehyde **2** and cytochrome C. At the same time, the protein secretion of Bax, p53, CC-3 and CC-9 was reduced, and the cell cycle and process of apoptosis was changed (Figure 7), so it can be speculated that benzaldehyde **2** has the effect of inhibiting apoptosis in HT-22 cells by blocking caspases family-related pathways. Due to its small molecular size, it can be assumed that the compound is highly bioavailable and has the potential to cross the blood–brain barrier (BBB) to reach microglia and neurons in the brain [37]. Structurally, benzaldehyde **2** contains an unsaturated double bond, whereas benzaldehyde **1** does not. Many studies have shown that polyunsaturated fatty acids containing two or more unsaturated double bonds have antioxidant effects, and can act as neuroprotective molecules that contribute to the survival of nerve cells [38,39]. For example, DHA promotes the aggregation of phosphatidylserine, which contributes to the migration and phosphorylation of protein kinases, which inhibit caspase-3 activity and prevent further neuronal apoptosis. Furthermore, combined with the experimental results of this study, it can be inferred that benzaldehyde **2** exhibits stronger anti-inflammatory, antioxidant and neuroprotective activities due to the presence of unsaturated bonds. Given all this, it can be hypothesized that benzaldehyde **2** may have an ameliorative effect on AD.

## 4. Materials and Methods

### 4.1. Material 

**Figure 10:**
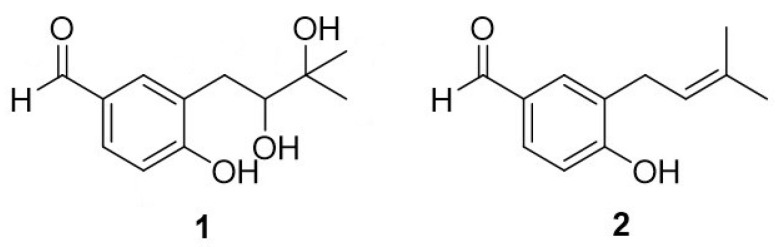
The structures of benzaldehydes **1**–**2**.

In the previous study, we purified and identified two benzaldehydes (Figure 10), which came from the coral fungus *Aspergillus terrestris* C23-3, isolated from Xuwen Nature Reserve in the South China Sea [18,19,21]. Benzaldehyde **1** (Terreusol, (*S*)-3-(2,3-dihydroxy-3-methylbutyl)-4-hydroxybenzaldehyd): ^1^H and ^13^C NMR data (in CD_3_OD): δ_H_ 7.67 (1H, d, *J* = 2.0 Hz, H-2), 6.89 (1H, d, *J* = 8.3 Hz, H-5), 7.66 (1H, dd, *J* = 8.4, 2.0 Hz, H-6), 3.11 (1H, dd, *J* = 16.7, 5.0 Hz, H_a_-1′), 2.81 (1H, dd, *J* = 16.7, 6.9 Hz, Hb-1′), 3.82 (1H, dd, *J* = 6.9, 5.1 Hz, H-2′), 9.78 (1H, s, H-7); δ_C_ 130.98 (C-1), 134.01 (C-2), 122.02 (C-3), 160.50 (C-4), 118.75 (C-5), 192.99 (C-7), 69.70 (C-2′), 79.90 (C-3′), 21.73 (C-4′), 25.86 (C-5′). Benzaldehyde **2** (4-hydroxy-3-(3-methylbut-2-en-1-yl)-benzaldehyde): ^1^H and ^13^C NMR data (in CD_3_OD): δ_H_ 7.61 (1H, d, *J* = 2.0 Hz, H-2), 6.87 (1H,d, *J* = 8.2 Hz, H-5), 7.59 (1H, dd, *J* = 8.2, 2.1 Hz, H-6), 9.70 (1H, s, H-7), 3.32 (2H, d, *J* = 7.4 Hz, H_2_-1′), 5.32 (1H, m, H-2′), 1.75 (3H, s, H_3_-4′), 1.71 (3H, s, H_3_-5′); δ_C_ 131.54 (C-1), 129.94 (C-2), 130.50 (C-3), 163.55 (C-4), 116.08 (C-5), 134.01 (C-6), 193.12 (C-7), 28.94 (C-1′), 122.94 (C-2′), 132.26 (C-3′), 25.94 (C-4′), 17.82 (C-5′).

Dulbecco’s modified Eagle’s medium (DMEM), fetal bovine serum (FBS), phosphate-buffered saline (PBS) and trypsin (0.25%) were purchased from Gibco (Carlsbad, CA, USA). Lipopolysaccharide (LPS), Beta-Amyloid 25–35 (Aβ25-35), dimethyl sulfoxide (DMSO) and 2′,7′-dichlorofluoresin diacetate (DCFH-DA) were purchased from Sigma Chemical (St. Louis, MO, USA). The monoclonal antibodies and secondary antibodies were provided by Santa Cruz Biotechnology Inc. (Santa Cruz, CA, USA). Other chemicals which were used were of analytical grade and are commercially available. 

### 4.2. Cell Culture

BV-2 and HT-22 cell lines were purchased from the Cell Bank of the Chinese Academy of Sciences (Shanghai, China). Both were cultured in DMEM, 10% FBS and 1% penicillin/streptomycin in a humidified incubator of 5% CO_2_ at 37 °C.

### 4.3. Cytotoxic Assessment

BV-2 or HT-22 cells were seeded in a 96-well plate before being incubated in culture medium, with different concentrations (0, 0.1, 1 and 10 μM) of two benzaldehydes (**1** and **2**), for 1 h, respectively. Subsequently, the BV-2 cells were stimulated by LPS (1 μg/mL) for 24 h, while HT-22 cells were incubated with Aβ (10 μM) for 48 h. Then, 10 μL of CCK−8 was added to each well for 1 h. Absorbance was measured by spectrophotometry (BioTek, Winooski, VT, USA) at a 540 nm wavelength.

### 4.4. IL-6, TNF-α and IL-18 Determination

The levels of IL-6, TNF-α and IL-18 in BV-2 cells were detected by using an enzyme-linked immunosorbent assay kit (ELISA). Briefly, cells were cultured in 24-well plates for 24 h before being treated with different concentrations (0, 0.1, 1 and 10 μM) of two benzaldehydes (**1** and **2**), respectively, for 1 h, and then were stimulated by LPS (1 μg/mL). After 24 h, the culture supernatant fluid was collected and centrifuged at 1000× *g* for 20 min. The levels of IL-6, TNF-α and IL-18 in the supernatant of cultured BV-2 cells were detected by using an ELISA kit (Elabscience, Wuhan, China). The optical density (OD) at 450 nm was detected in a microplate reader.

### 4.5. Western Blotting

Cell lysates were acquired using RIPA buffer with a protease and phosphatase inhibitor cocktail after being rinsed thrice with cold PBS. Protein expression was measured as described previously [30]. Briefly, 8–15% sodium dodecyl sulfate-polyacrylamide gel electrophoresis (SDS-PAGE) was carried out to separate the protein before transferring it onto nitrocellulose (NC) filter membranes (Amersham, CA, USA). The membranes were blocked with 5% skim milk, dissolved by TBST (Tris-buffered saline with 0.1% Tween-20), at room temperature for 2 h, followed by incubation with primary antibody at 4 °C overnight. Subsequently, membranes were washed three times and incubated with secondary antibody with HRP at room temperature for 2 h. Images obtained with blots were quantified by chemiluminescence apparatus and analyzed by image J.

### 4.6. ROS Determination

The accumulation of ROS in HT-22 cells was assessed by a DCFH-DA assay kit (Beyotime, Shanghai, China). Briefly, after stimulation by Aβ with different concentrations (0, 0.1, 1 and 10 μM) of two benzaldehydes (**1** and **2**) treatment, respectively, cells were immediately washed twice with phosphate-buffered saline (PBS) and loaded with a fluorescent probe (10 μM DCFH−DA, 30 min). Intracellular ROSs were evaluated using a fluorescence microscope (Olympus, Japan) and Image J.

### 4.7. Measurement of Mitochondrial Membrane Potential

The mitochondrial membrane potential in HT-22 cells were assessed by a Mitochondrial Membrane Potential Assay Kit with JC-1 (Solarbio, China). Briefly, HT-22 cells were cultured in 24-well plates for 24 h. They were treated with different concentrations (0, 0.1, 1 and 10 μM) of two benzaldehydes (**1** and **2**), respectively, for 1 h, and either exposed to Aβ or not. After being washed twice with PBS, cells were loaded with JC-1 for 20 min and washed with JC-1 staining buffer. The mitochondrial membrane potential was measured using a fluorescence microscope (Olympus, Japan).

### 4.8. Cell Cycle and Apoptosis Analysis

The cell cycle and apoptosis were measured using cell cycle and apoptosis analysis kit (Beyotime, China). Briefly, HT-22 cells, cultured in 24-well plates, were respectively treated with different concentration (0, 0.1, 1, and 10 μM) of two benzaldehyde (**1** and **2**), respectively, and Aβ for 24 h. They were washed two times and incubated with Propidium Iodide (PI) and Annexin V (AV) for 10 min at room temperature. The cell cycle and apoptosis were evaluated by a fluorescence microscope (Olympus, Japan).

### 4.9. Molecular Docking

The structure of benzaldehyde **2** was drawn using ChemBioDraw Ultra 14.0 (Figure 10) and converted into a 3D structure using ChemBio3D Ultra 14.0, which was then optimized using MMFF94 force field. The receptor protein used for this docking was downloaded from the RCSB Protein Data Bank (www.rcsb.org, accessed on 29 August 2022) to obtain the 3D structure of cytochrome C (PDB ID:5DF5). Both cytochrome C and compound benzaldehyde **2** were converted using AutodockTools 1.5.6 into PDBQT format. In this project, Autodock vina 1.1.2 was used for molecular docking studies. Based on the position of the ligand, the coordinates of the cytochrome C active site were determined as: center-x = 15.345, center-y = 11.387, center-z = 7.122; size-x = 40, size-y = 40, size-z = 40. To increase the accuracy of the calculation, we set the parameter exhaustiveness to 100. The default values are used for all parameters, except where noted. Finally, the conformation with the highest scoring value was selected for analysis of the results using PyMoL 1.7.6.

### 4.10. Statistical Analysis

All experiments were performed in at least triplicate, and data were expressed as the means ± SD (*n* = 3). Differences between multiple groups were analyzed by one-way analysis of variance (ANOVA) and *t*-test. A value of *p* < 0.05 was considered statistically significant.

## 5. Conclusions

**Figure 11:**
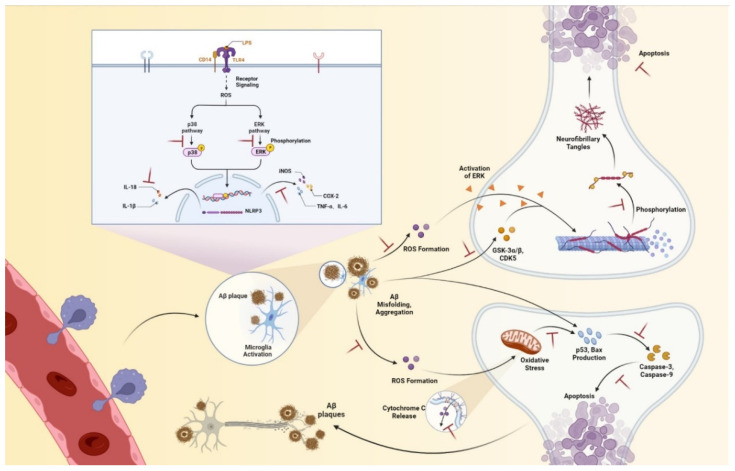
The mechanisms of two benzaldehydes improved neuroinflammation, and benzaldehyde **2** reduced neuronal damage. Benzaldehydes **1**-**2** reduced the LPS-induced neuroinflammation via feasible signaling mechanisms. They also inhibited the protein level expression of iNOS and COX-2, the release of TNF-α, IL-6, IL-18, and levels of phosphorylated ERK and p38. Benzaldehyde **2** down-regulated the Aβ-stimulated neuronal damage via caspase family-related pathways. In addition, it inhibited the protein level expression of CDK5 and GSK-3α/β, and reduced phosphorylation of Tau. Benzaldehyde **2** also reduced the release of ROS and further alleviated the decrease in mitochondrial membrane potential, with less cytochrome C flowing out.

In conclusion, we provide the first comprehensive description of the therapeutic potential of benzaldehyde **2** in Alzheimer’s disease, including amelioration of neuroinflammation and subsequent remission of neuronal damage, via reducing the secretion of inflammatory mediators and proinflammatory factors by microglia, and ultimately attenuating neuronal damage (Figure 11). These ameliorative effects of benzaldehyde **2** may be related to the inhibition of MAPKs signaling pathway, ROS, caspase family-related pathways and Tau phosphorylation. Our research provides new insights into LPS-induced neuroinflammation, as well as Aβ-induced neuronal protection mechanisms, aiming to delay the progression of AD. In addition, benzaldehyde **2** appears to have potential as a therapeutic agent for Alzheimer’s disease.

## Data Availability

The authors confirmed that the data supporting the findings of this study are available within the article.
